# Trend and early clinical outcomes in patients with preexisting atrial fibrillation undergoing isolated mitral valve surgery with or without surgical ablation

**DOI:** 10.1177/02676591251361352

**Published:** 2025-07-14

**Authors:** Jeremy Chan, Saifullah Mohamed, Gianni D Angelini

**Affiliations:** 1 1980 Bristol Medical School, University of Bristol, Bristol, UK; 2 156594 Department of cardiac surgery, Bristol Heart Institute, Bristol, UK

**Keywords:** mitral valve repair, surgical atrial fibrillation ablation, atrial fibrillation, mitral valve replacement, mitral valve surgery

## Abstract

**Introduction:**

European and American guidelines recommend concomitant surgical ablation for atrial fibrillation (AF) in patients undergoing mitral valve surgery. There is evidence that this intervention reduces the incidence of early and mid-term incidence of AF post-operatively. We aim to report the trend and early clinical outcomes in this cohort of patients in the United Kingdom.

**Method:**

This study included all patients with underlying atrial fibrillation who underwent first-time, elective, or urgent isolated mitral valve repair/replacement from 2011 to April 2019. We evaluated the trend and early clinical outcomes between patients who did/did not receive surgical AF ablation and examined associated factors.

**Results:**

A total of 3497 patients were included, with a median age of 70.6 years old (IQR: 63.1, 76.5), and 52.67% were male. The number of isolated mitral valve surgery performed ranges between 388 to 464 during the study period. The mitral valve repair rate was 62%. The overall AF ablation rate was 27.71% (Range: 16.74%-33.33%). After inverse propensity score matching, patients who underwent AF ablation had a significantly longer cardiopulmonary bypass (125 vs 99 mins, *p* < .001) and aortic cross-clamp time (92 vs 73 mins, *p* < .001). However, there was no difference in in-hospital mortality (2.03% vs 1.80%, *p* = .69), return to theatre for bleeding (5.82% vs 7.44%, *p* = .11), post-operative stroke (0.61% vs 0.48%, *p* = .11), post-operative dialysis (2.54% vs 2.40%, *p* = .83) and deep sternal wound infection (0.56% vs 0.88%, *p* = .34).

**Conclusion:**

Patients with pre-existing atrial fibrillation undergoing concomitant surgical ablation during mitral valve intervention had a longer cardiopulmonary bypass and cross-clamp time without compromising short-term clinical outcomes. Long-term outcomes are required to examine the potential lasting benefit of surgical ablation.

## Introduction

The incidence of patients undergoing cardiac surgery with a past medical history of Atrial fibrillation (AF) is reported as 4–10% in the current literature.^1,2^ Pre-operative AF is an independent risk factor for all-cause mortality, ischaemic stroke and heart failure.^1,2^ Surgical AF ablation aims to restore sinus rhythm to avoid the use of long-term anticoagulation and reduce the incidence of thromboembolism. The 2024 European Heart Rhythm Association/Heart Rhythm Society/Asia Pacific Heart Rhythm Society/Latin American Heart Rhythm Society and the American Society of Thoracic Surgeons have a Class I recommendation to perform concomitant AF ablation during mitral valve surgery in patients with pre-operative atrial fibrillation.^3,4^

However, the adoption and early clinical outcomes in patients with pre-operative AF undergoing mitral valve surgery with/without concomitant AF ablation are less reported. To our knowledge, this has not been reported in the UK. We aim to present the trend and early outcomes in this cohort of patients using a national United Kingdom database.

## Method

All patients with pre-operative AF who underwent first-time, elective or urgent isolated mitral valve repair/replacement for degenerative mitral valve disease from 2011 to 2019 were included in the National Cardiac Surgical Audit (NACSA) database. The NACSA database prospectively collects data on all major heart operations carried out on National Health Service patients in the UK since April 1996. The definitions of database variables used and a description of the database was previously reported.^5^ Patients who had previous cardiac surgery, emergency or salvage surgery, acute infective endocarditis, pre-operative sinus rhythm and concomitant procedures were excluded from this study. A flow chart diagram is included in the supplementary file.

Patients were then divided into two groups: (1) mitral valve intervention and surgical AF ablation and (2) Mitral valve intervention only. After inverse probability treatment weighting (IPTW), the trend and early post-operative clinical outcomes, including in-hospital mortality, the incidence of postoperative cerebral vascular accident, new haemodialysis, and deep sternal wound infection, were compared.

### Ethical statement

The study was part of a research project approved by the Health Research Authority and Health and Care Research Wales. As the study included retrospective interrogation of the NICOR database, the need for individual patient consent was waived (Health and Care Research Wales) (IRAS ID: 278171) in accordance with the research guidance. The study was performed in accordance with the ethical standards as laid down in the 1964 Declaration of Helsinki and its later amendments.

### Statistical analysis

Continuous and Categorical variables were reported as median and Interquartile range (IQR) as well as frequencies and percentages, respectively. The normality of the data was assessed using the Shapiro-Wilk test. Pearson’s Chi-squared test, Wilcoxon rank-sum test and one-way/multi-factor analysis of variance were used to compare two categorical variables, for comparison between means of two continuous, independent samples and to compare between 3 continuous variables, respectively. After IPTW, McNemar’s Chi-squared test was used to compare categorical variables.

**Figure 1.**
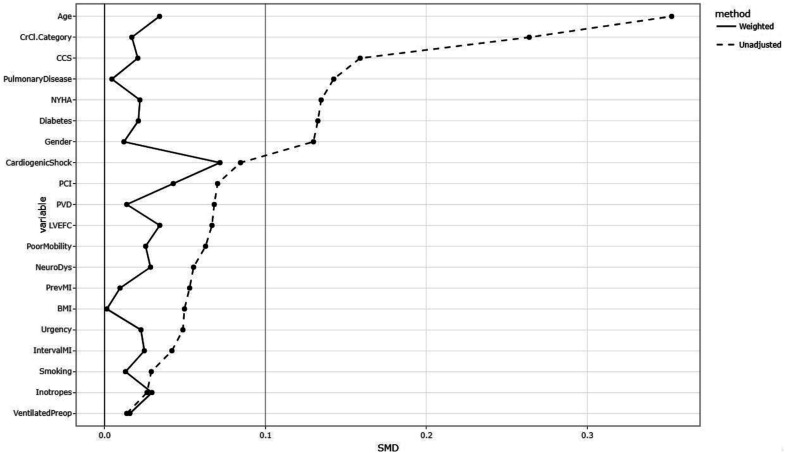
Figure 1 shows the standardised mean difference before and after inverse propensity score matching.

IPTW was performed to create two balanced groups before direct comparison. The effectiveness of IPTW in balancing covariates across two classes was previously described.^6^ In addition, the advantages of IPTW over propensity score matching, in studies with a small number of events and/or a large number of confounders were shown by Chesnaye et al.^7^ The effectiveness of IPTW before and after was evaluated using the standardised mean difference (SMD) between groups. A SMD of less than 0.1 is considered to be adequate. Figure 1 shows the SMD of pre-operative characteristics before and after IPTW.

Binary logistic regression was performed using the baseline patient demographics and comorbidities to predict factors associated with the use of surgical AF ablation in the whole cohort. Pearson correlation coefficient was used to identify if there is a correlation between the number of isolated mitral valve surgeries performed in cardiac surgical units and the percentage of patients who received surgical AF ablation. R (Version 4.2.3, R Foundation for Statistical Computing, Vienna, Austria) and R Studio (Version 1.4.1103, RStudio, PBC) were used to perform statistical analysis. Graphs and tables were created using R (Version 4.2.3, R Foundation for Statistical Computing, Vienna, Austria) and Microsoft Office 365 (Version 16.0.14026, Microsoft Corporation, Washington, U.S.).

## Results

**Figure 2.**
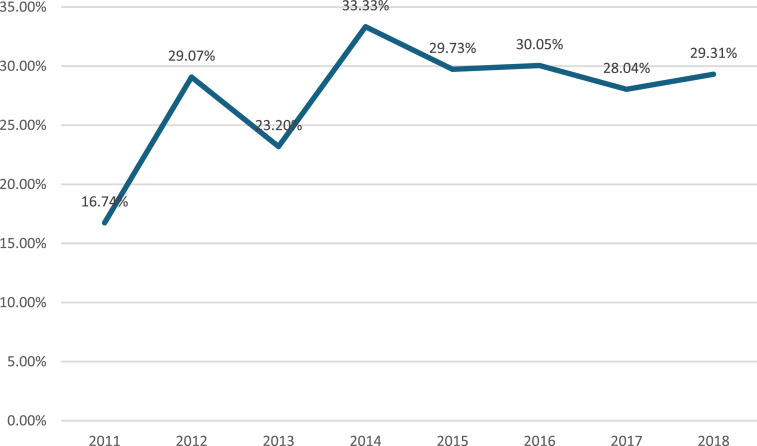
Figure 2 shows the proportion of patients with pre-operative atrial fibrillation undergoing isolated mitral valve intervention and receiving surgical AF ablation.

A total of 3497 patients were included in the study, of which 969 (27.71%, ranging from16.74% to 33.33%) underwent concomitant AF ablation (Figure 2). The median age of the cohort was 70.6 years old (IQR: 63.1, 76.5 years old), and 52.67% were male. The number of isolated mitral valve surgeries performed ranged between 388 and 464 during the study period. The mitral valve repair rate was 62%.

### Clinical outcomes between patients with/without concomitant AF ablation

Before IPTW, patients who underwent concomitant AF ablation were younger than those who did not receive AF ablation 67.8 (IQR: 60.8, 73.6) versus 71.7 (IQR: 64.3, 77.5) years old, *p* < .001). In addition, there was a high proportion of male (66% vs 50%, *p* < .001), less history of pre-operative pulmonary disease (90% vs 85%, *p* < .001), and diabetes (94% vs 90%, *p* = .011) when compared the mitral valve with concomitant AF ablation and mitral valve intervention alone group. Table 1 shows the pre-operative characteristics before and after IPTW.

**Table 1.** Pre-operative characteristics in patients with preexisting atrial fibrillation undergoing isolated mitral valve surgery with/without surgical atrial fibrillation ablation before and after IPTW.

Pre-operative characteristics	Whole cohort Pre-IPTW			Post IPTW			
	No AF ablation (*n* = 2528)	AF ablation (*n* = 969)	*p*-value	No AF ablation (Weight: 3503.89)	AF ablation (Weight 3447.21)	SMD	*p*-value
Age (yrs median [IQR])	71.7 [64.3, 77.5]	67.8 [60.8, 73.6]	<0.001	70.7 [62.7, 76.7]	70.0 [63.4, 75.2]	0.03	0.06
Gender (Female)	1267 (50.1%)	423 (43.7%)	<0.001	1691.5 (48.3)	1643.3 (47.7)	0.01	0.76
BMI (median [IQR])	26.2 [23.5, 29.5]	26.5 [23.9, 29.4]	0.06	26.2 [23.5, 29.6]	26.3 [23.6, 29.2]	0.001	0.53
LVEFC			0.42			0.03	0.79
Good (LVEF > 50%)	1778 (70.3)	661 (68.2)		2443.4 (69.7)	2399.7 (69.6)		
Moderate (LVEF 31%–50%)	151 (6.0)	68 (7.0)		219.1 ( 6.3)	220.7 ( 6.4)		
Poor (LVEF 21%–30%)	597 (23.6)	240 (24.8)		839.4 (23.9)	826.9 (23.9)		
Very Poor (LVEF <21%)	2 ( 0.08)	0 (0.00)		2.00 ( 0.1)	0.00 ( 0.00)		
Urgent operation	294 (11.6)	98 (10.1)	0.23				
Diabetes			0.01			0.02	0.97
Not diabetic	2278 (90.1)	908 (93.7)		3194.2 (91.1)	3160.9 (91.7)		
Diet control	69 ( 2.7)	18 ( 1.9)		86.8 ( 2.5)	82.6 ( 2.4)		
Oral therapy	147 ( 5.8)	35 ( 3.6)		181.2 ( 5.2)	167.8 ( 4.9)		
Insulin therapy	34 ( 1.3)	8 ( 0.8)		41.7 ( 1.2)	35.9 ( 1.0)		
Smoking			0.746			0.01	0.95
Never smoked	1398 (55.30)	531 (54.80)		1930.8 (55.1)	1888.6 (54.8)		
Ex smoker	1005 (39.75)	395 (40.76)		1405.5 (40.1)	1400.7 (40.6)		
Current smoker	125 ( 4.94)	43 ( 4.44)		167.5 ( 4.8)	157.9 ( 4.6)		
PVD	86 ( 3.40)	22 ( 2.27)	0.105	108.8 ( 3.1)	115.5 ( 3.4)	0.01	0.76
Neuro dys	74 ( 2.93)	20 ( 2.06)	0.195	95.1 ( 2.7)	110.3 ( 3.2)	0.03	0.54
CrCl. Category			<0.001			0.02	0.98
Normal (CC > 85 mL/min)	513 (20.29)	109 (11.25)		620.7 (17.7)	591.9 (17.2)		
Moderate (CC 50-85 mL/m)	1137 (44.98)	494 (50.98)		1638.9 (46.8)	1635.3 (47.4)		
Severe (CC < 50 mL/min)	860 (34.02)	364 (37.56)		1224.1 (34.9)	1199.1 (34.8)		
CCS class			0.003			0.02	0.99
0	1947 (77.02)	801 (82.66)		2752.6 (78.6)	2702.79 (78.4)		
1	306 (12.10)	76 ( 7.84)		383.7 (10.9)	385.67 (11.2)		
2	204 ( 8.07)	68 ( 7.02)		272.3 ( 7.8)	265.61 ( 7.7)		
3	57 ( 2.25)	21 ( 2.17)		78.8 ( 2.25)	80.89 ( 2.4)		
4	14 ( 0.55)	3 ( 0.31)		16.5 ( 0.5)	12.24 ( 0.4)		
NYHA class			0.005			0.02	0.96
1	176 ( 6.96)	83 ( 8.57)		258.9 ( 7.4)	264.73 ( 7.7)		
2	882 (34.89)	386 (39.83)		1272.8 (36.3)	1267.27 (36.8)		
3	1283 (50.75)	432 (44.58)		1717.9 (49.0)	1680.97 (48.8)		
4	187 ( 7.40)	68 ( 7.02)		254.3 ( 7.3)	234.24 ( 6.8)		
PCI			0.43			0.04	0.73
No previous PCI	2426 (95.97)	939 (96.90)		3370.7 (96.2)	3312.7 (96.1)		
PCI < 24 hours before surgery	6 ( 0.24)	1 ( 0.10)		7.01 ( 0.2)	6.54 ( 0.2)		
PCI > 24 hours before surgery; same admission	93 ( 3.68)	29 ( 2.99)		3.00 ( 0.1)	0.00 ( 0.0)		
Previous MI			0.79			0.02	0.89
None	2514 (99.45)	962 (99.28)		3481.2 (99.4)	3426.45 (99.4)		
One	1 ( 0.04)	0 ( 0.00)		1.00 ( 0.03)	0.00 ( 0.00)		
Two or more	8 ( 0.32)	5 ( 0.52)		14.8 ( 0.4)	14.86 ( 0.4)		
Poor mobility	99 ( 3.92)	27 ( 2.79)	0.133	127.1 ( 3.6)	142.05 ( 4.1)	0.03	0.58
Ventilated reop	6 ( 0.24)	3 ( 0.31)	0.98	8.9 ( 0.3)	6.29 ( 0.2)	0.02	0.65
Cardiogenic shock	9 ( 0.36)	0 ( 0.00)	0.10	9.00 ( 0.3)	0.00 ( 0.00)	0.07	0.003
Inotropes	12 ( 0.47)	3 ( 0.31)	0.70	14.6 ( 0.4)	8.5 ( 0.3)	0.03	0.41

AF: Atrial fibrillation, CCS: Canadian Cardiovascular Society, PCI: Percutaneous Coronary Intervention, NYHA: New York Heart Association, LMS: Left Main stem disease, MI: Myocardial infraction, LVEF: Left ventricular ejection fraction, NeuroDys: Neurological Dysfunction, ES2: Euro Score II, IPTW: Inverse probability treatment weighting, SMD: Standardised mean difference, PVD: Peripheral vascular disease, CrCl.category: Creatinine clearance category, Ventilated Pre op: Require invasive ventilation (including intubation) pre-operatively, Inotropes: Inotropic support prior to general anaesthesia.

After IPTW generated two balanced groups, patients who underwent AF ablation had a significantly longer cardiopulmonary bypass (median: 125 mins, IQR: 103,154 mins vs 99 mins, IQR: 80, 123 mins, *p* < .001) and aortic cross-clamp time (92 mins, IQR: 73,115 mins vs 73 mins, IQR: 58, 91 mins, *p* < .001). However, there was no difference in in-hospital mortality (2.03% vs 1.80%, *p* = .69), return to theatre for bleeding (5.82% vs 7.44%, *p* = .11), post-operative stroke (0.61% vs 0.48%, *p* = .11), post-operative dialysis (2.54% vs 2.40%, *p* = .83) and deep sternal wound infection (0.56% vs 0.88%, *p* = .34) when comparing patients receiving isolated mitral intervention and those with concomitant surgical AF ablation.

Patients who received concomitant AF ablation had a significantly higher rate of repair rate (72.34% vs 49.61%, *p* < .001) (Table 2).

**Table 2.** Intra- and post-operative outcomes in patients with preexisting atrial fibrillation undergoing isolated mitral valve surgery with/without surgical atrial fibrillation ablation before and after IPTW.

Characteristics	Whole cohort Pre-IPTW			Post IPTW		
Variable	No AF ablation (*n* = 2528)	AF ablation (*n* = 969)	*p*-value	No AF ablation (Weight: 3503.89)	AF ablation (Weight 3447.21)	*p*-value
Mitral valve intervention			<0.001			<0.001
Replacement	1166 (46.1%)	245 (25.3%)		1606.1 (45.8%)	893.2 (25.9%)	
Repair	1362 (63.9%)	724 (74.7%)		1897.8 (54.2%)	2554.0 (74.1%)	
CPB (mins) (Median, IQR)	98 [79, 122]	126 [104, 156]	<0.0001	99 [80, 123]	125 [103, 154]	<0.0001
XClamp time (mins) (Median, IQR)	73 [58, 91]	94 [75, 117]	<0.0001	73 [58, 91]	92 [73, 115]	<0.0001
Mortality	54 ( 2.2)	15 ( 1.6)	0.30	69.85 ( 2.03)	62.00 (1.80)	0.69
RTT	136 ( 5.9)	62 ( 6.9)	0.33	185.26 ( 5.82)	236.66 (7.44)	0.11
Postop CVA	14 ( 0.6)	4 ( 0.5)	0.16	19.89 ( 0.61)	14.70 ( 0.48)	0.11
Postop dialysis	66 ( 2.8)	20 ( 2.2)	0.42	83.99 ( 2.54)	77.88 ( 2.40)	0.83
Postop DSWI at discharge	10 ( 0.6)	8 ( 1.0)	0.32	13.93 ( 0.56)	24.78 ( 0.88)	0.34

CPB: Cardiopulmonary bypass time, RTT: Return to theatre, CVA: Cerebrovascular accident, IPTW: Inverse probability treatment weighting, DSWI: Deep sternal wound infection, XClamp: Aortic Cross clamp.

### Factors predicting the use of AF ablation

Older patients (OR: 0.97 95% CI: 0.96-0.98, *p* < .001), patients with pre-operative pulmonary disease (OR:0.71, 95% CI: 0.55–0.91, *p* = .008) and diabetes requiring oral hypoglycaemics (OR: 0.59 95% CI: 0.44–0.97, *p* = .034 ) were less likely to receive surgical AF ablation.

Patients with mildly impaired creatinine clearance levels were more likely to undergo surgical AF ablation (OR: 1.54, 95%CI: 1.20-1.97, *p* = .001) (Table 3).

**Table 3.** Table 3 shows the factors predicting the use of surgical atrial fibrillation ablation.

Characteristics	OR	95% CI	*p*-value
Age (years)	0.97	0.96–0.98	<0.001
Gender (Female)	0.87	0.74–1.02	0.01
BMI	1.01	0.99–1.02	0.31
Good LV function	Reference		
Moderate LV function	1.55	0.55–1.91	0.98
Poor LV function	2.55	0.65–5.38	0.97
Very poor LV function	5.75	0.46–19.28	0.96
Urgent operation	0.9	0.69–1.18	0.44
No diabetes	Reference		
Diabetes (Diet control)	0.73	0.42–1.24	0.24
Diabetes (Oral therapy)	0.66	0.44–0.97	0.03
Diabetes (Insulin)	0.59	0.27–1.32	0.20
Non smoker			
Ex-smoker	1.11	0.94–1.30	0.22
Current smoker	0.79	0.53–1.15	0.22
Pulmonary disease	0.71	0.55–0.91	0.01
Peripheral vascular disease	0.86	0.53–1.41	0.56
Neurological dysfunction	0.8	0.29–2.16	0.66
Normal creatinine clearance level	Reference		
Mildly impaired creatinine clearance level	1.54	1.20–1.97	0.01
Moderately impaired creatinine clearance level	1.11	0.83–1.49	0.47
Severely impaired creatinine clearance level	0.43	0.09–1.96	0.28
CCS class 0	Reference		
CCS class 1	0.65	0.49–0.85	0.002
CCS class 2	0.88	0.65–1.18	0.39
CCS class 3	1.04	0.62–1.76	0.88
CCS class 4	0.73	0.20–2.72	0.64
NYHA class 1	Reference		
NYHA class 2	1.05	0.78–1.41	0.74
NYHA class 3	0.9	0.67–1.21	0.48
NYHA class 4	1.06	0.70–1.61	0.77
No previous PCI	Reference		
Previous PCI	0.37	0.04–3.16	0.37
PCI < 24 hours before surgery	0	0.00–1.00	0.98
PCI > 24 hours before surgery; same admission	0.91	0.55–1.48	0.670
No previous MI	Reference		
Previous MI: One episode	0.91	0.52–1.58	0.73
Previous MI: >=2 episode	0.65	0.17–2.44	0.52
Poor mobility	0.89	0.38–2.10	0.79
Ventilated preoperatively	2.55	0.46–14.28	0.29
Cardiogenic shock	0	0.00–1.00	0.96
Inotropes use preoperatively	0.64	0.15–2.70	0.55

### Unit volume and proportion of patients undergoing surgical AF ablation

**Figure 3.**
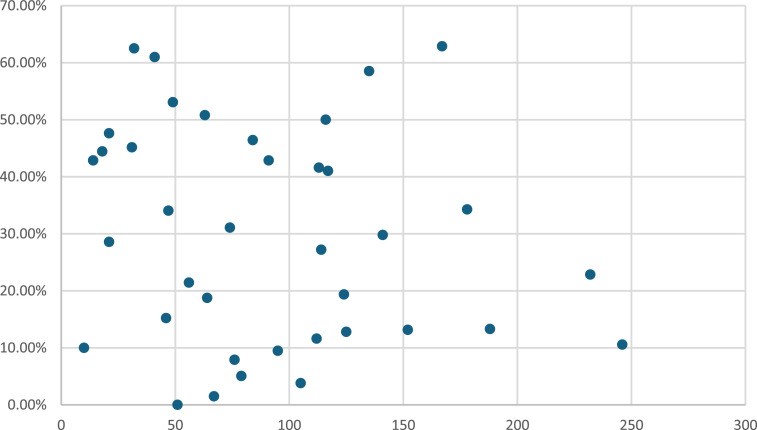
Figure 3 shows the volume of isolated mitral valve surgery performed between 2011 and 2019 (*x*-axis) and the proportion of patients undergoing surgical AF ablation (*y*-axis) in all cardiac surgical centres in the UK.

Of the 38 cardiac surgical centres included during the study period, the median number of AF ablation performed was 20 (IQR: 8.25, 37.25) cases, ranging from 0% to 62.87% of patients undergoing mitral valve intervention (Figure 3).

There was no correlation between the total number of isolated mitral valve surgeries performed and the percentage of patients who received surgical AF ablation from the centre level (R = −0.18, *p* = .28).

## Discussion

Our results demonstrated that concomitant surgical AF ablation in patients undergoing mitral valve surgery can be performed safely without an increase in-hospital mortality and early complications, despite a longer cardiopulmonary bypass and aortic cross-clamp time. The adoption of surgical AF ablation in the UK has been consistently around 30%, with variation in unit adoption from 0% to 63%.

Surgical AF ablation during cardiac surgery has been shown to improve freedom from AF at 12 months.^1^ In addition, surgical AF ablation did not increase mortality, stroke and myocardial infarction.^1^ This allows the potential cessation of anticoagulation in patients with sustained sinus rhythm and evidential follow-up through ambulatory monitoring and CT evidence of complete left atrial appendage occlusion. The 2024 European Heart Rhythm Association/Heart Rhythm Society/Asia Pacific Heart Rhythm Society/Latin American Heart Rhythm Society expert consensus statement on catheter and surgical ablation of atrial fibrillation has advised to perform surgical AF ablation in patients with paroxysmal or persistent AF undergoing left atrial open cardiac surgery, regardless of prior antiarrhythmic drug failure or intolerance.^3^ The guideline has also recommended that surgeons perform a Biatrial Cox maze procedure or a minimum of pulmonary vein isolation plus left atrial posterior wall isolation during left atrial surgery.^3^ In addition, The Society of Thoracic Surgeons 2023 Clinical Practice Guidelines for the Surgical Treatment of Atrial Fibrillation has a class IA recommendation for Surgical ablation to be performed during first-time nonemergent concomitant mitral operations, intending to restore sinus rhythm and improve long-term outcomes.^4^

The adoption of surgical ablation in the United Kingdom is less than other reports in the literature. Data from the US have shown an average ablation rate of 48.3% and up to 68.4% for mitral valve (+/−CABG) surgery.^8^ A study conducted by Belley-Cote showed that only 20% of patients received AF ablation in an international, multi-institutional survey. They have also reported a median number of 10 ablations performed by individual surgeons annually, with marked variation in proportions of patients with AF considered for ablation.^9^

Similar to all surgical procedures, surgical AF ablation does carry its risks. A randomised control trial by Gillinov and colleagues demonstrated a three-fold increase per 100 patient-years (21.5 vs 8.1 per 100 patient-years, *p* = .001) in pacemaker implantation in patients who had undergone surgical AF ablation during mitral valve surgery.^10^ This was significantly higher than the incidence of 5%–10% reported from the US registry data.^11^ One explanation could be due to the fact that ∼50% of the surgical ablation group had multivalve surgery, which increases the risk of atrioventricular block.^10^ In addition, the study by Lee and colleagues suggested that pacemaker implantation within the first year after concomitant mitral valve surgery and Cox-maze procedure does not significantly impact long-term mortality or ischaemic stroke risk.^12^ In our study, there was no increased incidence of mortality or major early postoperative complications.

Training in surgical AF ablation in the UK also varies; not all programs offer ablation. It is not part of the national curriculum, and there are no fellowship programmes. A study by Deboard et al. demonstrated that residents in the US have performed a median number of five ablations during residency, with nearly 80% of residents unable to perform Cox-MAZE IV procedures independently.^13^ It is hoped that the promotion of fellowships, such as the Atrial Fibrillation Fellowship by the European Association for Cardiothoracic Surgery, would promote the use of this technique.

This study has several limitations. Most notably, the lack of short-term pre-discharge rhythm and long-term follow-up data prevented us from assessing the success rate of AF ablation, the incidence of sinus rhythm and thromboembolism, which are key benefits of the procedure. The technique used for AF ablation, MAZE versus pulmonary vein isolated and the energy source Radiofrequency and Cryoablation, were not included in the database. Additionally, the absence of data on pacemaker implantation rates and pre-operative atrium diameter limited our ability to evaluate the procedure’s associated risks. all retrospective registry studies, this study is susceptible to selection bias. Additionally, we did not assess individual surgeon and hospital volume or experience, which could influence outcomes. The accuracy of registry data depends on healthcare professionals’ compliance, and missing data may introduce bias. However, data for EuroScore II variables and postoperative status at discharge are mandatory, ensuring completeness in these aspects. Lastly, the details of atrial fibrillation (Biatrial/Left-sided Cox MAZE procedure/Pulmonary vein isolation) were not captured in the database.

Using propensity score matching, such as IPTW, can introduce limitations, including residual confounding due to unmeasured variables and potential model misspecification. Additionally, IPTW relies on large sample sizes to achieve stable weight estimation, and extreme weights can lead to increased variability in results. However, IPTW offers several advantages, including the ability to balance observed covariates between treatment groups, thereby reducing confounding and improving causal inference. It also allows for including the entire study population rather than restricting analysis to matched pairs, preserving statistical power.

## Conclusion

Concomitant surgical ablation in patients with preexisting atrial fibrillation undergoing mitral valve surgery increased cardiopulmonary bypass and aortic cross-clamp time. However, the procedure can be performed safely, with no increase in mortality and early in-hospital complications. Further studies are required to examine the successful rate of AF ablation and the long-term outcomes, including the incidence of stroke and the requirement for anticoagulation use.

Data availability statement: The data underlying this article were provided by the National Institute for Cardiovascular Outcomes Research under licence / by permission. Data will be shared on request to the corresponding author with permission of the National Institute for Cardiovascular Outcomes Research.
